# Design and Synthesis of Novel Xyloketal Derivatives and Their Protective Activities against H_2_O_2_-Induced HUVEC Injury

**DOI:** 10.3390/md13020948

**Published:** 2015-02-12

**Authors:** Shixin Liu, Rong Luo, Qi Xiang, Xianfang Xu, Liqin Qiu, Jiyan Pang

**Affiliations:** 1 School of Chemistry & Chemical Engineering, Sun Yat-Sen University, Guangzhou 510275, China; E-Mails: lsxinhoho@gmail.com (S.L.); gdzdlr@163.com (R.L.); xuxianf@mail.sysu.edu.cn (X.X.); qiuliqin@mail.sysu.edu.cn (L.Q.); 2 Institute of Biomedicine & Guangdong Provincial Key Laboratory of Bioengineering Medicine, Jinan University, Guangzhou 510632, China; E-Mail: txiangqi@jnu.edu.cn; 3 Department of Pharmacy, Jinan University, Guangzhou 510632, China

**Keywords:** Xyloketals, H_2_O_2_, Oxidative stress, 3D-QSAR, HUVECs

## Abstract

In this work, we designed and synthesized a series of amide derivatives (**1**–**13**), benzoxazine derivatives (**16**–**28**) and amino derivatives (**29**–**30**) from xyloketal B. All 28 new derivatives and seven known compounds (**14**, **15**, **31**–**35**) were evaluated for their protection against H_2_O_2_-induced HUVEC injury. **23** and **24** exhibited more potential protective activities than other derivatives; and the EC_50_ values of them and the leading compound **31** (xyloketal B) were 5.10, 3.59 and 15.97 μM, respectively. Meanwhile, a comparative molecular similarity indices analysis (CoMSIA) was constructed to explain the structural activity relationship of these xyloketal derivatives. This 3D QSAR model from CoMSIA suggested that the derived model exhibited good predictive ability in the external test-set validation. Derivative **24** fit well with the COMSIA map, therefore it possessed the highest activity of all compounds. Compounds **23**, **24** and **31** (xyloketal B) were further to examine in the JC-1 mitochondrial membrane potential (MMP) assay of HUVECs using flow cytometry (FCM). The result indicated that **23** and **24** significantly inhibited H_2_O_2_-induced decrease of the cell mitochondrial membrane potential (ΔΨm) at 25 μM. Collectively, the protective effects of xyloketals on H_2_O_2_-induced endothelial cells may be generated from oxidation action by restraining ROS and reducing the MMP.

## 1. Introduction

Cardiovascular disease (CVD) has drawn significant attention in recent years because it has become the leading cause of mortality worldwide, affecting people from every income level. Reactive oxygen species (ROS), including H_2_O_2_, OH^−^, NO, and ONOO^−^, play key roles in the pathogenesis of many CVDs, such as hypertension and atherosclerosis (As). The ROS-induced oxidative stress in cardiac and vascular is closely connected with the endothelial dysfunction in disease initiation and progression. Reactive oxygen species (ROS) are generated under pathological conditions, such as ischemia-reperfusion and inflammation, and activate pro-apoptotic and anti-apoptotic signaling programs in endothelial cells [1]. As one of the most common ROS, hydrogen peroxide (H_2_O_2_) can easily cross the plasma membrane, produce a highly reactive radical OH·, and lead to cell and tissue damage [2,3]. The generation of H_2_O_2_ plays a key role in the atherosclerotic progression. H_2_O_2_ mediates various cellular responses. Direct or indirect stimulation by H_2_O_2_ due to its intracellular production could activate various cellular pathways, including calcium release, protein tyrosine kinase, mitogen-activated protein kinases (MAPKs), transcription factor NF-κB, and the induction of cell apoptosis [4,5,6,7]. Thus, H_2_O_2_ has been extensively used as an oxidative stimulus to induce oxidative stress in *in vitro* models. As the major type of endothelial cells, human umbilical vein endothelial cells (HUVECs) are commonly accepted as a model cell to explore the mechanisms involved in the pathogenesis of CVDs [8].

Mitochondrion serve as a pivotal decision center in many types of apoptotic response: they release a variety of death-promoting factors from their inter-membrane spaces into the cytosol, triggering an increase in mitochondria permeability and leading to consequences of mitochondrial dysfunction (e.g., disruption of the mitochondrial membrane potential ΔΨm) [9,10]. Mitochondria are considered the main source of ROS in the cell. Unless adequately detoxified, superoxide causes mitochondrial oxidative stress and may contribute to a decline in mitochondrial function.

**Chart 1:**
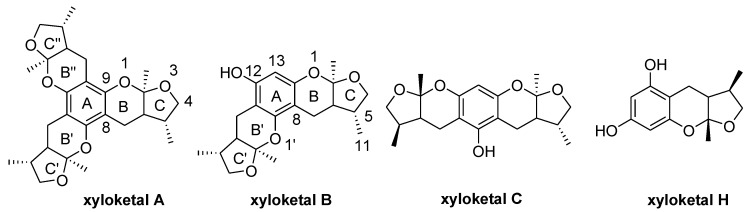
Structures of xyloketal A, B, C, H.

**Chart 2:**
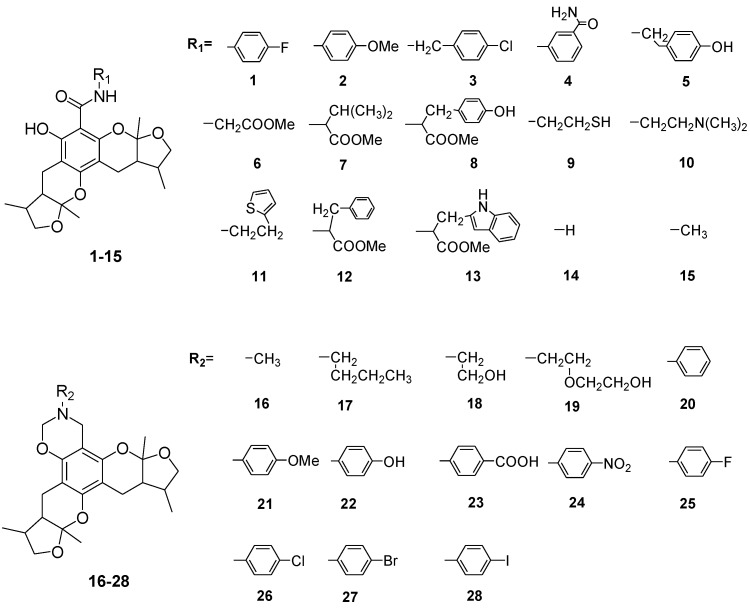

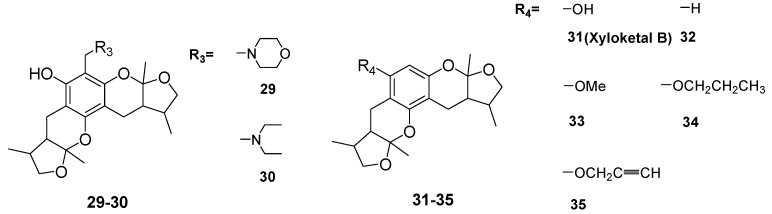
Structures of xyloketal derivatives **1**–**35**.

Xyloketals are a type of novel compounds that possess unique molecular structures. They are isolated from the marine mangrove fungus *Xylaria* sp. (#2508) (Chart 1) [11,12]. We previously demonstrated that xyloketal B has protective action against a variety of pathophysiological stimuli, such as oxLDL, oxygen-glucose deprivation (OGD) and 1-methyl-4-phenylpyridinium (MPP+), in different disease models [13,14,15,16,17,18]. Thus, xyloketal B might be a good candidate for further development as an antioxidant medicine in cardiovascular diseases. However, its clinical development may be difficult due to water insolubility. Structure-activity relationship analyses in previous reports have demonstrated that the characteristic substituted groups at the C-12 or C-13 position of xyloketal B are key functional groups for its antioxidative effect. To improve the solubility and biological activity of xyloketal B, some amino groups can be introduced at the C-12 or C-13 position of this type of structure, and the corresponding acid salts could be prepared in the future. Because of the complexity of the stereoselective synthesis of xyloketals, it is difficult to provide a significant amount of optically pure samples for biological activity evaluation. We decided to begin the studies using racemic xyloketal B. In this paper, we designed and synthesized a new series of derivatives (Chart 2) from xyloketal B, including a series of C-13 xyloketal amide derivatives (**1**–**13**); xyloketal benzoxazine derivatives (**16**–**28**) using a one-pot reaction of xyloketal B, formaldehyde and different primary amines; and xyloketal amino derivatives (**29**–**30**) that C-13 substituted using different secondary amines. All 28 new derivatives and 7 known compounds (**14**, **15**, **31**–**35**) were evaluated for their protection against H_2_O_2_-induced HUVEC injury. Then, a comparative molecular similarity indices analysis (CoMSIA) was constructed using the SYBYL programming package (version 7.3.5) to explain the structural activity relationship of these xyloketal derivatives [19,20]. The training set and test set were randomly divided out of a total of 35 molecules. A training set of 30 molecules was used to construct the QSAR model, and a training set of five molecules was used to validate it. Mitochondria are considered the main source of reactive oxygen species (ROS) in cells [21,22]. Therefore, we investigated whether xyloketals could protect mitochondria through inhibition of ROS. Any compound with high antioxidative action was further investigated in the JC-1 mitochondrial membrane potential (MMP) assay of HUVECs using flow cytometry (FCM).

## 2. Results and Discussion

### 2.1. Chemistry

**Scheme 1:**
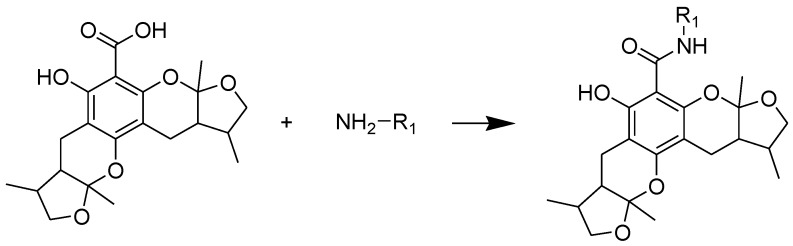
Synthesis of compounds **1**–**13**. Reagents and conditions: BOP, DIEA, DMF, room temp.

**Scheme 2:**
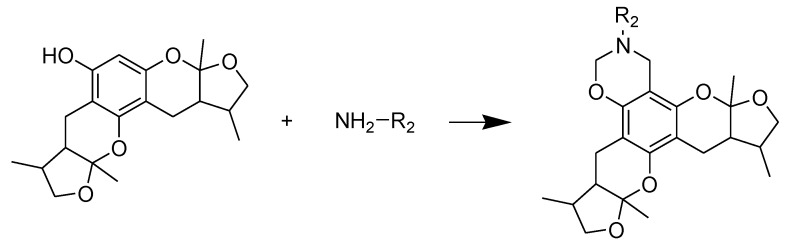
Synthesis of compounds **16**–**28**. Reagents and conditions: THF, HCHO, room temp.

**Scheme 3:**
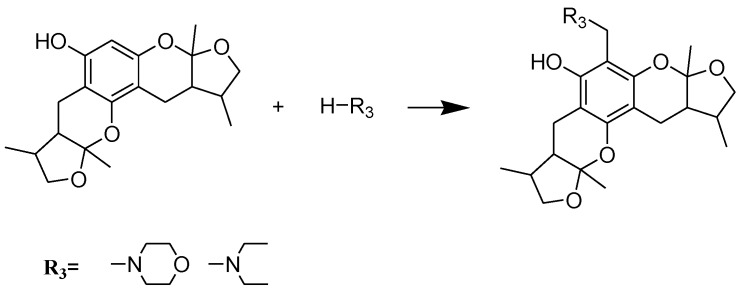
Synthesis of compounds **29**–**30**. Reagents and conditions: THF, HCHO, room temp.

**Chart 3:**
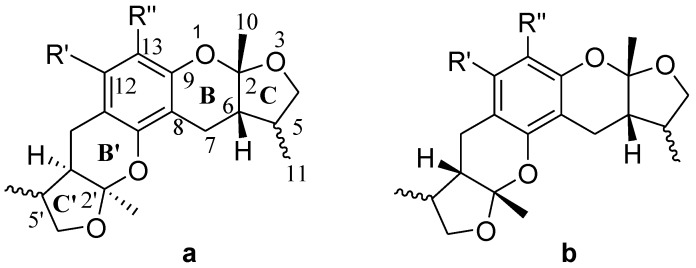
All stereoisomers of synthesized xyloketal structures.

**Figure 1:**
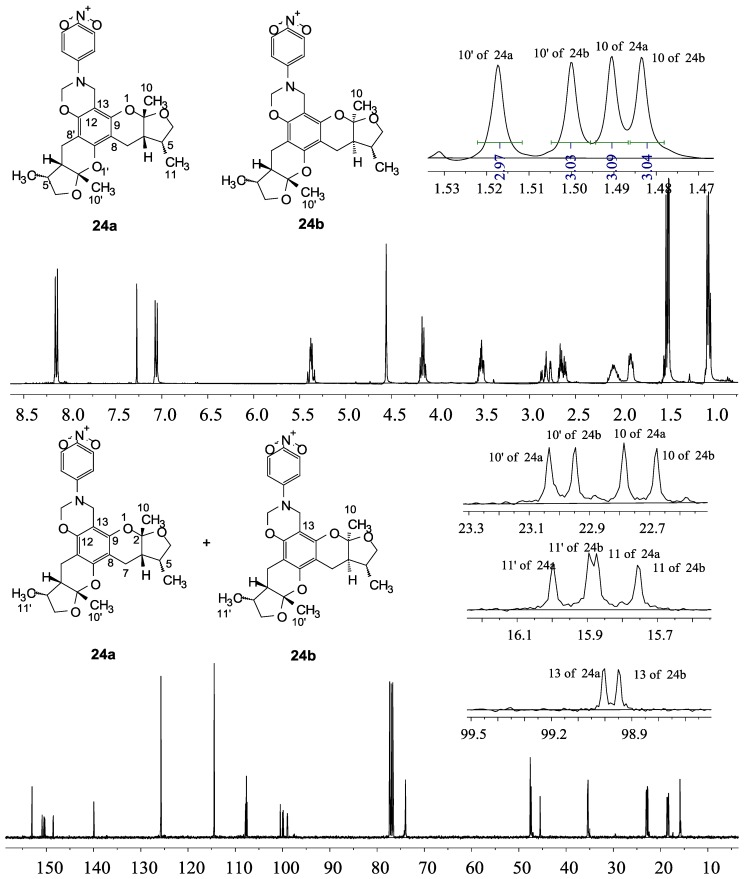
^1^H and ^13^C NMR spectra of **24**.

The general synthetic routes of compounds **1**–**35** are outlined in Scheme 1, Scheme 2 and Scheme 3. All the new compounds were prepared from xyloketal B and xyloketal B acid that were gained from synthetic way in the ordinary state without any asymmetric factors [16]. New xyloketal amides **1**–**13** were obtained via a condensation reaction between xyloketal B acid and the corresponding amines in the presence of (benzotriazol-1-yloxy)tris(dimethylamino)phosphonium hexafluorophosphate (BOP) and *N*,*N*-diisopropylethylamine (DIEA) (Scheme 1). Interestingly, the one-pot Mannich reaction of xyloketal B, formaldehyde and different primary amines afforded a series of novel xyloketal derivatives **16**–**28** bearing an 1,3-oxazine moiety. Instead of primary amines, the Mannich reaction of secondary amines, xyloketal B and formaldehyde generated C-13 substituted amine derivatives **29**–**30** (Scheme 3). All the new compounds were fully characterized using MS and NMR. Moreover, all of the examined compounds were synthesized as racemic mixtures from synthetic xyloketal B and xyloketal B acid and no asymmetric synthesis was applied. Their stereo features are the same as xyloketal B and xyloketal B acid. The stereochemistry of these xyloketal derivatives was complicated. In principle, the two oxygen-containing pyran and furan rings B and C can be connected in a *cis* or *trans* fashion. The methyl group at C-5 or C-5′ could be *cis* or *trans* with respect to the stereogenic centers at the junction at C-2 or C-2′ and C-6 or C-6′. However, previous studies indicated that rings B and C or B′ and C′ were *cis* for all condensations leading to xyloketal derivatives in the natural and synthetic compounds [18,23,24,25,26,27,28,29], thus only two sets of stereoisomers of xyloketals can be formed: *syn*, *anti* and *syn*, *syn* types. Moreover, C-2/C-5 methyl in *cis* orientation occupied dominant position both in experimental and theoretical results [29]. We previously also reported that synthetic xyloketal B and xyloketal B acid were characterized as mixtures of stereoisomers, including the enantiomers and diastereoisomers [16,18,30], and the ratio of two sets of diastereoisomers *syn*, *anti* and *syn*, *syn* was about 1:1 via NMR analysis. Similar to our previous studies, at this time, racemic mixtures of all new derivatives were consisted of two sets of diastereoisomers (Chart 3, *syn*, *anti* **a** and *syn*, *syn* **b**). Every diastereoisomer had four enantiomer pairs depending on C-5/C-5′ methyl in *cis* or *trans*, and the isomer with C-2/C-5 and C-2′/C-5′ methyl all in *cis* may take greatest proportion in these four enantiomer pairs in according to previous studies. The very close relationship of the diastereoisomers *syn*, *anti* and *syn*, *syn* was evident in NMR spectra. Though with overlapping of nearly identical sets of signals, two sets of signal peaks could still be detected in ^1^H and ^13^C NMR spectra assigned to isomers *syn*, *anti* and *syn*, *syn* with approximate ratio of ~1:1. Taking **24** as an example, both the ^1^H and ^13^C NMR spectra of **24** showed evidence of the diastereoisomers **24a** and **24b** (Figure 1). Obviously, in ^1^H NMR, methyl (10 and 10′) at C-2 and C-2′ showed as two peaks respectively (δ *=* 1.49, 1.48, and 1.52, 1.50 ppm) relative to two single peak (δ *=* 1.50 and 1.52 ppm) of 10 and 10′ in the natural xyloketal B [11] (Figure 1A), in addition, the integrals of the hydrogen atoms of two peaks indicated an approximately 1: 1 ratio of diastereoisomers **24a** and **24b**. The ^13^C NMR spectrum was more instructive (Figure 1B). The methyl (C-10, C-10′) and (C-11, C-11′) both presented as four closely packed peaks (δ *=* 23.0, 22.9, 22.8, 22.6 and 16.0, 15.9, 15.9, 15.8 ppm). Moreover, the aromatic carbon atom (C-13) also appeared as two peaks (δ *=* 98.9 and 99.0 ppm). These peaks all proved that compound **24** consisted of two sets of diastereoisomers. However, the enantiomers could not be found by NMR analysis because of their identical NMR spectra. Separating these stereoisomers via chromatography was very difficult. Therefore, all xyloketal derivatives were used directly in the biological screening without separating the stereoisomers this time. These compounds possessed the same structural framework; the only differences were different substituents at the C-12 or C-13 position of the aromatic ring. Although the test compounds are enantiomeric and diastereomeric mixtures, their activities and SAR analysis could be obtained.

### 2.2. Xyloketal Derivatives Protected Endothelial Cells against H_2_O_2_-Induced Injury Assay

**Figure 2:**
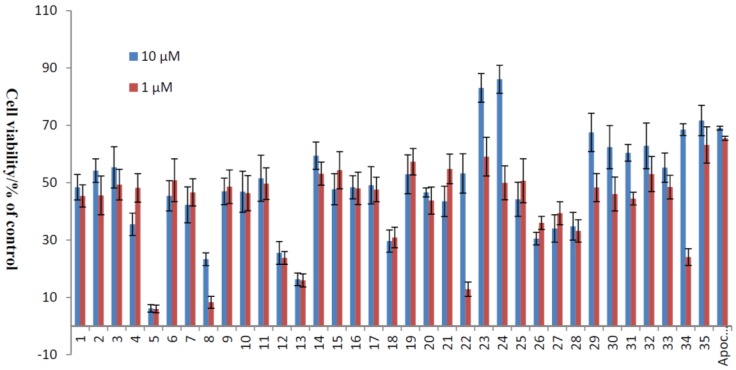
Protective effects of xyloketal derivatives against H_2_O_2_-induced cell injury. HUVECs were pre-incubated with 10 μM xyloketal derivatives (blue bars) or 1 μM xyloketal derivatives (red bars) for 30 min, and 600 μM H_2_O_2_ was added to the medium. After incubation for 20 h, cell viability was determined using MTT reduction assay. Apocynin was used as positive control. Values are the mean ± SD (*n* = 6).

The apoptosis of HUVECs caused by ROS has been implicated in numerous pathophysiological processes of CVDs. An important source of endogenous ROS is generated from H_2_O_2_, and it has been proven that ROS are involved in the apoptosis of ECs [31,32]. Using a similar culture system, we have shown that xyloketals had no significant effects on cell viability up to 100 μM in the MTT assay. Accordingly, the protection of xyloketals **1**–**35** at concentrations of 1 and 10 μM was applied for the following H_2_O_2_ (600 μM)-induced injury of HUVECs, and apocynin (1 and 10 μM) was used as a positive control. The results (Table 1, Figure 2) showed that some compounds exhibited strong antioxidative activities, in both morphological changes and inhibition of cell apoptosis. Among them, benzo-1,3-oxazine xyloketal derivatives **23** and **24** displayed greater potential protective activities than other derivatives with cell viabilities 83.07% and 86.08%. Furthermore, to evaluate the activities of these two most significant compounds clearly, the EC_50_ values in HUVECs ranged from 1–50 μM of **23**, **24** and the leading compound xyloketal B (**31**) were determined with 5.10, 3.59 and 15.97 μM, respectively (Table 2). Thus, new candidates with amino groups, which could be prepared the corresponding acid salts in the future to improve their water-insolubility, will be the promising compounds for further evaluation in the treatment of cardiovascular diseases.

**Table 1:** Protective effects of xyloketal derivatives against H_2_O_2_-induced cell injury.

No.	Cell Viability/% of Control		No.	Cell Viability/% of Control	
	10 μM	1 μM		10 μM	1 μM
**1**	48.41 ± 4.47	45.38 ± 3.89	**19**	52.94 ± 6.80	57.32 ± 4.59
**2**	54.19 ± 4.11	45.57 ± 6.78	**20**	46.63 ± 1.55	43.77 ± 4.70
**3**	55.36 ± 7.21	49.33 ± 5.34	**21**	43.49 ± 5.28	54.78 ± 5.16
**4**	35.49 ± 3.90	48.19 ± 4.96	**22**	53.23 ± 6.86	12.85 ± 2.53
**5**	6.15 ± 1.29	5.96 ± 1.36	**23**	83.07 ± 5.01	59.07 ± 6.76
**6**	45.41 ± 5.29	50.84 ± 7.46	**24**	86.08 ± 4.87	49.95 ± 5.92
**7**	42.28 ± 6.27	46.64 ± 4.76	**25**	44.20 ± 5.95	50.67 ± 7.66
**8**	23.32 ± 2.22	8.29 ± 2.08	**26**	30.47 ± 2.19	35.97 ± 2.28
**9**	46.98 ± 4.63	48.61 ± 5.84	**27**	34.02 ± 4.76	39.33 ± 4.00
**10**	46.84 ± 7.17	46.34 ± 6.13	**28**	34.79 ± 4.82	33.18 ± 3.92
**11**	51.56 ± 8.03	49.66 ± 5.51	**29**	67.53 ± 6.68	48.30 ± 4.91
**12**	25.51 ± 3.94	23.76 ± 2.24	**30**	62.41 ± 7.52	46.04 ± 5.92
**13**	16.31 ± 2.19	15.91 ± 2.30	**31**	60.43 ± 2.89	44.46 ± 2.24
**14**	59.42 ± 4.76	53.14 ± 4.03	**32**	62.85 ± 7.96	53.00 ± 6.14
**15**	47.72 ± 5.41	54.35 ± 6.44	**33**	55.24 ± 5.09	48.48 ± 4.13
**16**	48.40 ± 4.02	48.01 ± 5.62	**34**	68.50 ± 2.06	24.06 ± 2.87
**17**	49.11 ± 6.50	47.62 ± 4.30	**35**	71.67 ± 5.28	63.16 ± 6.32
**18**	29.64 ± 3.88	30.88 ± 3.59	**Apo-cynin**	69.03 ± 0.68	65.48 ± 0.70

Datas are representative of means ± S.E.M. *n* = 6 wells for each group.

**Table 2:** The EC_50_ of **23**, **24** and **31** (xyloketal B).

The EC_50_ of **23**, **24** and **31** (xyloketal B)			
No.	**23**	**24**	**31** (xyloketal B)
EC_50_^a^ (μM)	5.10	3.59	15.97

^a^ Each experiment was independently performed six times and expressed as means.

### 2.3. The Structural Activity Relationship of Xyloketals on a COMSIA Model

**Chart 4:**
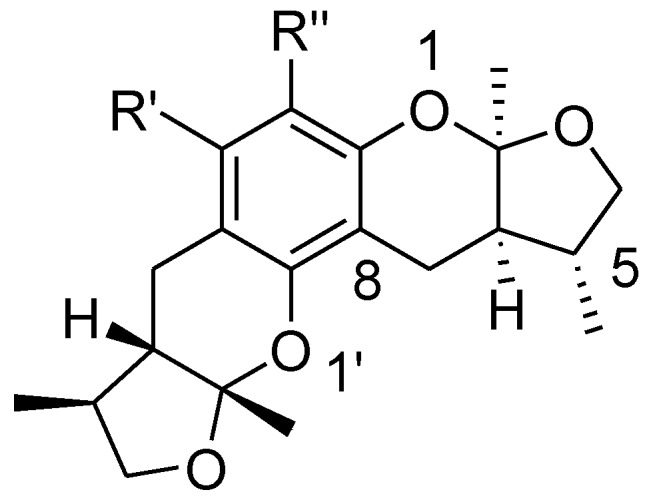
A stereoisomer of synthesized xyloketal structure used to 3D SAR analysis.

To explore the SAR of these xyloketals, a COMSIA model was constructed to explain the structural activity relationship of xyloketal B and its analogs. These compounds had the same structural framework, to unify the evaluation standard; therefore, a dominating stereoisomer of a previously reported xyloketal structure was selected for use in this SAR analysis (Chart 4) [16,18,30]. The statistical parameters of the 3D-QSAR models are shown in Table 3. For an acceptable standard of a 3D-QSAR model, the *q*^2^ training (cross-validated regression coefficient) of the training set should be greater than 0.5, and the *r*^2^ training (conventional regression coefficient) should be greater than 0.9. The LOO PLS analysis of the model gives a *q*^2^ value of 0.577 at six components, together with the conventional regression coefficient *r*^2^ of 0.988 and a standard error of estimate of 0.041.

**Table 3:** Statistical parameters of the CoMSIA models.

Training Set	
*q*^2^	0.577
*r*^2^	0.988
SEE ^a^	0.041
*F* ^b^	316.828
Optimal components	6
**Test set**	
*q*_test_^2^	0.648
*r*_test_^2^	0.858
*k*	0.987

^a^ Standard error of estimate; ^b^ *F*-test value.

**Figure 3:**
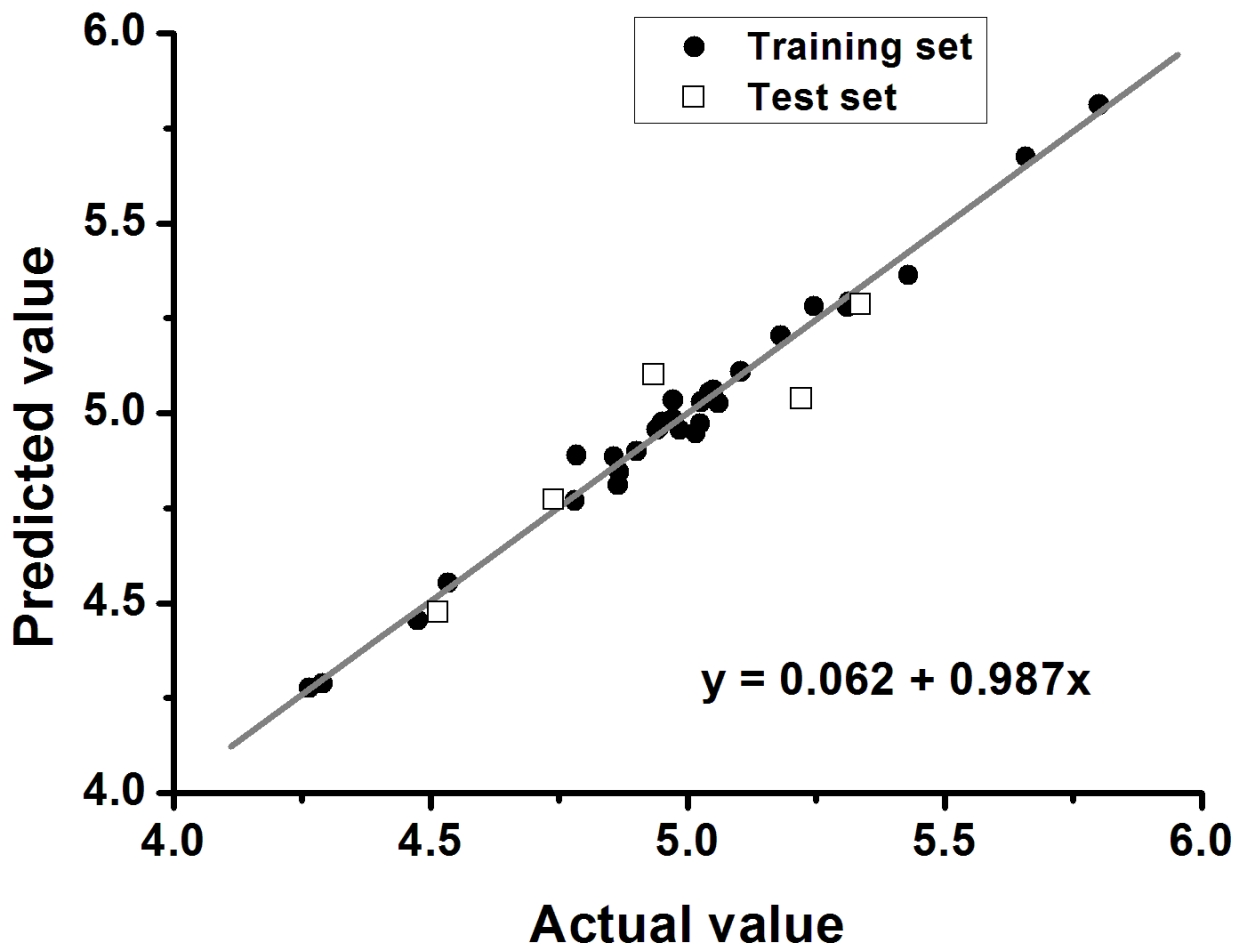
Plot of predicted *versus* experimental values of the 3D-QSAR CoMSIA model.

Furthermore, the significance and predictability of QSAR models should be further proven by an external test set using the following criteria: *q*^2^ > 0.5, *r*^2^ > 0.6 and 0.85 < *k* < 1.15 (k refers to the slope of the regression line between the experimental and the predicted biological activities). A graphical representation of the predicted and actual values is displayed in Figure 3. An excellent correlation between the experimental and predicted biological activities is shown in Figure 3 for the test set (*q*^2^ = 0.648, *r*^2^ = 0.858, and *k* = 0.987). In summary, all statistical data satisfied the recommended criteria, suggesting that the derived model exhibits good predictive ability in the external test-set validation. The predicted and actual values are shown for comparison in Table 4.

**Table 4:** Structures, experimental and predicted values of the xyloketal derivatives.

No.	Cell Viability/% of Control	Actual Value	Predicted Value	Residual Value
1	48.41 ± 4.47	4.972	4.984	−0.012
2	54.19 ± 4.11	5.026	5.029	−0.003
3	55.36 ± 7.21	4.901	4.900	0.001
4 *	35.49 ± 3.90	4.740	4.772	−0.032
5	6.15 ± 1.29	4.264	4.276	−0.012
6 *	45.41 ± 5.29	4.935	5.101	−0.166
7	42.28 ± 6.27	4.865	4.811	0.054
8 *	23.32 ± 2.22	4.515	4.476	0.039
9	46.98 ± 4.63	4.947	4.965	−0.018
10	46.84 ± 7.17	5.103	5.109	0.006
11	51.56 ± 8.03	4.857	4.886	−0.029
12	25.51 ± 3.94	4.534	4.553	−0.019
13	16.31 ± 2.19	4.290	4.288	0.002
14 *	59.42 ± 4.76	5.222	5.038	0.184
15	47.72 ± 5.41	5.060	5.026	0.034
16	48.40 ± 4.02	4.972	5.034	−0.062
17	49.11 ± 6.50	4.985	4.956	0.029
18	29.64 ± 3.88	4.475	4.454	0.021
19	52.94 ± 6.80	5.051	5.061	−0.010
20	46.63 ± 1.55	4.941	4.957	−0.016
21	43.49 ± 5.28	5.025	4.972	0.053
22	53.23 ± 6.86	4.951	4.976	−0.025
23	83.07 ± 5.01	5.658	5.674	−0.016
24	86.08 ± 4.87	5.801	5.811	−0.010
25	44.20 ± 5.95	5.016	4.948	0.068
26	30.47 ± 2.19	4.784	4.889	−0.105
27	34.02 ± 4.76	4.867	4.845	0.022
28	34.79 ± 4.82	4.781	4.770	0.011
29	67.53 ± 6.68	5.430	5.363	0.067
30	62.41 ± 7.52	5.042	5.054	−0.012
31	60.43 ± 2.89	5.181	5.204	−0.023
32	62.85 ± 7.96	5.311	5.280	0.031
33	55.24 ± 5.09	5.246	5.281	−0.035
34 *	68.50 ± 2.06	5.337	5.286	0.051
35	71.67 ± 5.28	5.314	5.293	0.021

* Molecules in the test set.

**Figure 4:**
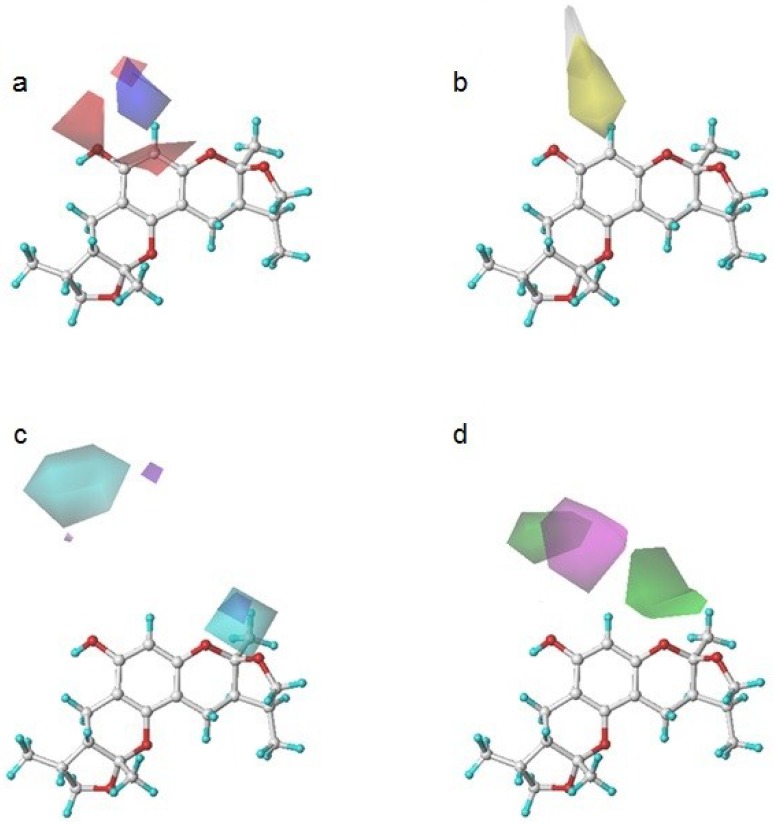
Xyloketal B in CoMSIA contour maps. (**a**) an electrostatic map: blue and red contour referred to regions where electropositive substituents were favorable and unfavorable for the compound activity; (**b**) a hydrophobic map: yellow and white contour referred to regions where hydrophobic substituents were favorable and unfavorable for the compound activity; (**c**) a hydrogen donor map: cyan and purple contour referred to regions where hydrogen donor substituents were favorable and unfavorable for the compound activity; (**d**) a hydrogen acceptor map: magenta and green contour referred to regions where hydrogen acceptor substituents were favorable and unfavorable for the compound activity.

To determine how to modify the structure of xyloketal B, we built a model using a series of derivatives to explain the structure-activity relationship. Figure 4 shows a contour map of each field in the presence of xyloketal B. These maps indicated the favorable and unfavorable modification of the compounds in the colored regions. They are (a) an electrostatic map highlighting the regions where electropositive components were favorable (shown in blue) and unfavorable (shown in red) for the activity; (b) a hydrophobic map highlighting the regions where hydrophobic components were favorable (shown in yellow) and unfavorable (shown in white) for the activity; (c) a hydrogen donor map highlighting the regions where hydrogen donor components were favorable (shown in cyan) and unfavorable (shown in purple) for the activity; and (d) a hydrogen acceptor map highlighting the regions where hydrogen donor components were favorable (shown in magenta) and unfavorable (shown in green) for the activity.

**Chart 5:**
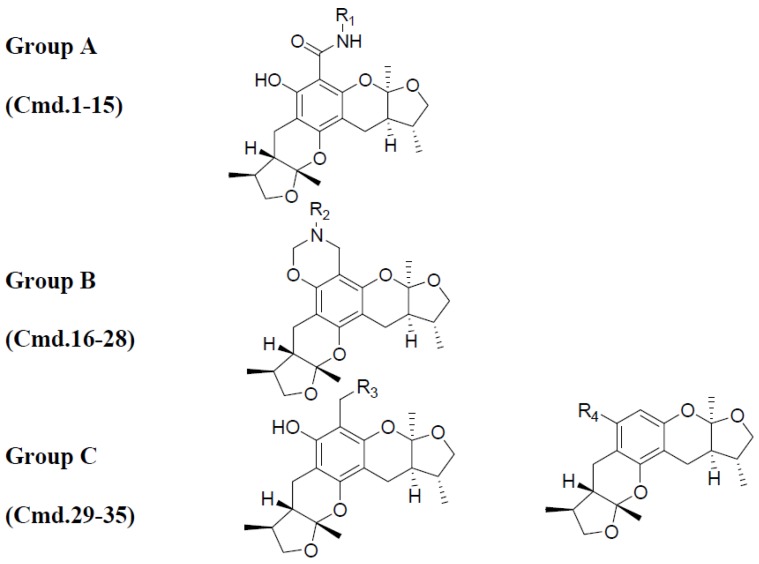
All 35 molecules were divided into three groups based on their structural similarities.

According to their structural discrepancy, all compounds can be cataloged into three groups. Their common structures are shown in Chart 5. Group A consisted of compounds **1**–**15** with activities ranging from 15.52 to 55.89. Group B consisted of compounds **16**–**28**, which had activities ranging from 22.97 to 86.34. It contained a unique 6-member ring with different substitution groups. Group C consisted of compounds **29**–**35**. No drastic modifications were made to these compounds. Therefore, their activities ranged from 52.41 to 72.89, similar to that of **31**-xyloketal B (60.29).

**Figure 5:**
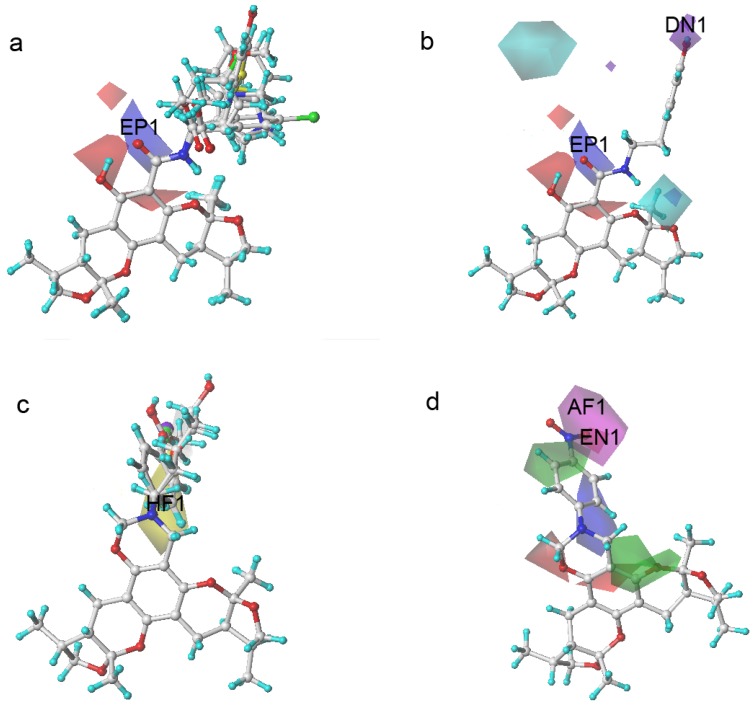
Xyloketal derivatives in CoMSIA contour maps. (**a**) compounds of Group A overlay with electrostatic maps; (**b**) compound **5** (cell viability % = 15.52) with electrostatic and hydrogen donor maps; (**c**) compounds of Group B overlay with hydrophobic maps; (**d**) compound **24** (cell viability % = 86.34) with electrostatic and hydrogen bond acceptor maps.

The compounds in Group A yielded relatively lower activities. This result could be explained by the mismatch of the CoMSIA force field with the substitution groups. As shown in Figure 5a, the overlay of the compounds in group A had a common amide group, and the oxygen fell into the blue area, EP1. As mentioned previously, the blue contour map indicates areas where positively charged components would increase the activity, but negatively charged components would decrease the activity. Take compound **5** as a more specific example; it not only had the aforementioned common amide group in EP1, but also an -OH group in the purple area DN1. Purple areas indicate that a hydrogen bond donor in the area would have a negative effect on the compound activity (Figure 5b). The above analysis justified why compound **5** had the lowest activity. Some of the compounds in Group B had stronger activities. An overlay of the compounds in Group B in Figure 5c revealed that the substitution groups of compounds in group B took a different orientation to fit in the yellow region HF1. Compound **24** extended its nitro group into the magenta area AF1 and the red area EN1, where hydrogen bond acceptor and electro-negative groups would elevate the activity of the compound, respectively. Compound **24** matched the CoMSIA map well (Figure 5d); therefore, it possessed the highest activity of all compounds. In contrast, compounds **25**–**28** had reduced activities due to replacing the nitro group with halogens, which were not hydrogen bond acceptors. Compounds in Group C produced similar activities to that of xyloketal B because no drastic structural changes were made to these compounds. The substitution groups did not specially fall into regions that increased or decreased the activities.

**Figure 6:**
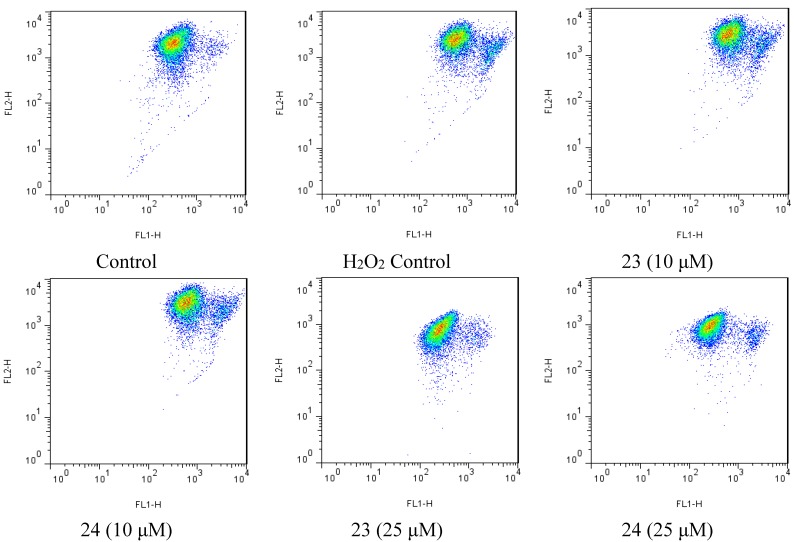
Flow cytometry scatter plot was analyzed using the Flowjo (v7.6.5), showing JC-1 monomers and aggregates in different group.

### 2.4. Xyloketal Derivatives Restored the H_2_O_2_-Induced Reduction of the Mitochondrial Membrane Potential (ΔΨm)

**Figure 7:**
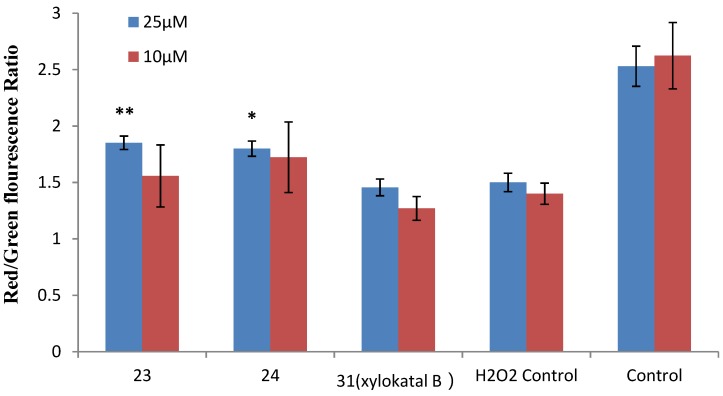
Effects of xyloketal B and the derivatives **23** and **24** on H_2_O_2_-induced decrease of ΔΨm in HUVECs. HUVECs were pre-incubated with 25 μM xyloketal derivatives (blue bars) or 10 μM xyloketal derivatives (red bars) for 30 min, and 400 μM H_2_O_2_ was added to the medium. After incubation for 20 h, ΔΨm was determined by FACS analyses. Values are the mean ± SD (*n* = 6): * *p* < 0.05, ** *p* < 0.01 *versus* H_2_O_2_.

Mitochondria are considered the main source of reactive oxygen species (ROS) in cells. Therefore, we investigated whether xyloketals could protect mitochondria via inhibition of ROS. Compounds **23** and **24** were examined in the JC-1 mitochondrial membrane potential (MMP) assay of HUVECs using flow cytometry (FCM). As shown in Figure 6 and Figure 7, **23** and **24** significantly inhibited the H_2_O_2_-induced the decrease in the cell mitochondrial membrane potential (ΔΨm) at 25 μM. Collectively, **23** and **24** effectively protected HUVECs against oxidative damage and further mitochondrial membrane integrity impairment and prevented H_2_O_2_-induced apoptosis of HUVECs by regulating the ROS-mediated mitochondrial dysfunction pathway.

## 3. Experimental Section

### 3.1. Chemistry

All reagents and solvents were of commercial quality and used without further purification. ^1^H and ^13^C NMR data were recorded on a Bruker AVANCE 400 MB NMR spectrometer (Bruker, Fallanden, Switzerland) operating at 400 and 101 MHz for ^1^H and ^13^C respectively. All chemical shifts are in ppm (δ) with respect to tetramethylsilane (TMS) as internal standard, and coupling constants (*J*) are in Hz. Mass spectra were obtained on DSQ (low resolution mass spectrometer) (Thermo, San Jose, CA, USA) and MAT95XP (high resolution mass spectrometer) instruments (Thermo, San Jose, CA, USA).

### 3.2. General Procedure of Synthesizing Compounds

**Compound 1**. First, 50 mg (0.13 mmol) of xyloketal B acid [16] and 22 mg (0.20 mmol) of *p*-fluoroaniline were dissolved in 10 mL of DMF in a 50 mL-round-bottom flask. Then, 80 mg (0.20 mmol) of BOP and 0.40 mL (2.0 mmol) of DIEA were added and stirred at room temperature overnight. The reaction was quenched with 10 mL saturated solution of ammonium chloride in ice water. The aqueous layer was extracted with ethyl acetate, 3 × 25 mL. The combined organic extracts were washed with saturated ammonium chloride and brine, dried over anhydrous MgSO_4_ and concentrated *in vacuo*. Purification by flash chromatography (petroleum ether: ethyl acetate = 5:1~2:1) gave the title compound. Yield 75%; white solid; m. p. 68–69 °C. ^1^H NMR (400 MHz,CDCl_3_) δ 13.97 (s, 1H), 10.59 (s,1H), 7.57 (d, *J* = 8.4 Hz, 2H), 7.03 (d, *J* = 8.4 Hz, 2H), 4.22–4.15 (m, 2H), 3.53–3.58 (m, 2H), 2.96–2.86 (m, 2H), 2.73–2.58(m, 2H), 2.14–2.08 (m, 2H), 2.02–1.93 (m, 2H), 1.61 (s, 3H), 1.53 (s, 3H), 1.09 (d, *J* = 6.0 Hz, 3H), 1.07 (d, *J* = 6.0 Hz, 3H). ^13^CNMR (101 MHz, CDCl_3_) δ 168.78. 161.96, 160.64, 158.22, 155.31, 150.93, 134.00, 122.44, 115.52, 110.57, 110.31, 108.43, 99.96, 98.40, 96.85, 74.63, 74.25, 47.84, 47.29, 35.68, 35.27, 23.51, 23.02, 18.61, 18.31, 15.89, 15.87. HR-EI-MS *m/z* 483.2057; calculated for C_27_H_30_FNO_6_: 483.2057.

**Compound 2**. The title compound was obtained from xyloketal B acid and *p*-anisidine with a procedure similar to that for compound **1**. Yield 73%; white solid; m. p. 70–71 °C. ^1^H NMR (400 MHz, CDCl_3_) δ 14.15 (s, 1H), 10.49 (s, 1H), 7.51 (d, *J* = 8.8 Hz, 2H), 6.90 (d, *J* = 8.8 Hz, 2H), 4.18 (dd, *J* = 8.4 Hz, 2H), 3.81 (s, 3H), 3.56 (dd, *J* = 8.4 Hz, 2H), 2.96–2.85 (m, 2H), 2.72–2.58 (m, 2H), 2.16–2.08 (m, 2H), 2.00–1.93 (m, 2H), 1.62 (s, 3H), 1.52 (s, 3H), 1.08 (d, *J* = 6.4 Hz, 3H), 1.07 (d, *J* = 6.4 Hz, 3H). ^13^C NMR (101 MHz, CDCl_3_) δ 168.62, 161.91, 156.51, 155.08, 151.00, 131.05, 122.56, 114.24, 110.42, 110.17, 108.50, 108.36, 99.89, 98.53, 97.03, 74.55, 74.24, 55.54, 47.77, 47.32, 35.77, 35.28, 23.14, 22.79, 18.75, 18.34, 16.05, 15.91. HR-EI-MS *m/z* 495.2251; calculated for C_28_H_33_NO_7_: 495.2257.

**Compound 3**. The title compound was obtained from xyloketal B acid and 4-chlorobenzylamine with a procedure similar to that for compound **1**. Yield 71%; white solid; m. p. 72–74 °C. ^1^H NMR (400 MHz, CDCl_3_) δ 14.18 (s, 1H), 8.93 (s, 1H), 7.30 (d, *J* = 8.4 Hz, 2H), 7.26 (d, *J* = 8.4 Hz, 2H), 4.59 (d, *J* = 2.0 Hz, 2H), 4.20–4.04 (m, 2H), 3.56–3.45 (m, 2H), 2.93–2.82 (m, 2H), 2.70–2.55 (m, 2H), 2.10–2.02 (m, 2H), 1.96–1.90 (m, 2H), 1.51 (s, 3H), 1.50 (s, 3H), 1.07 (d, *J* = 6.4 Hz, 3H), 1.05 (d, *J* = 6.4 Hz, 3H). ^13^C NMR (101 MHz, CDCl_3_) δ 170.58, 161.60, 155.01, 154.83, 151.40, 137.06, 132.93, 128.71, 128.54, 110.05, 109.83, 108.47, 99.70, 98.34, 96.68, 74.41, 74.20, 47.78, 47.37, 42.32, 35.72, 35.29, 23.07, 22.78, 18.69, 18.33, 16.04, 15.89. HR-EI-MS *m/z* 513.1907; calculated for C_28_H_32_ClNO_6_: 513.1918.

**Compound 4**. The title compound was obtained from xyloketal B acid and *m*-amino-benzamide with a procedure similar to that for compound **1**. Yield 78%; white solid; m. p. 108–109 °C. ^1^H NMR (400 MHz, CDCl_3_) δ 13.84 (s, 1H), 10.75 (s, 1H), 8.19 (s, 1H), 7.45 (m, 3H), 5.86 (br, 2H), 4.22–4.15 (m, 2H), 3.59–3.53 (m, 2H), 2.96–2.86 (m, 2H), 2.73–2.60 (m, 2H), 2.15–2.08 (m, 2H), 2.03–1.96 (m, 2H), 1.62 (s, 3H), 1.53 (s, 3H), 1.09 (d, *J* = 6.8 Hz, 3H), 1.07 (d, *J* = 6.8 Hz, 3H). ^13^C NMR (101 MHz, CDCl_3_) δ 169.10, 169.05, 162.01, 155.52, 150.98, 138.32, 134.37, 129.29, 124.14, 123.39, 119.47, 110.47, 108.49, 100.00, 98.57, 96.83, 74.67, 74.26, 47.86, 47.29, 35.76, 35.26, 23.23, 23.03, 18.71, 18.37, 16.04, 15.97. HR-EI-MS *m/z* 508.2201; calculated for C_28_H_32_N_2_O_7_: 508.2210.

**Compound 5**. The title compound was obtained from xyloketal B acid and tyramine with a procedure similar to that for compound **1**. Yield 72%; white solid; m. p. 97–98 °C. ^1^H NMR (400 MHz, CDCl_3_) δ 14.43 (s, 1H), 8.56 (s, 1H), 7.08 (d, *J* = 8.4 Hz, 2H), 6.76 (d, *J* = 8.4 Hz, 2H), 6.19 (s, 1H), 4.20–4.04 (m, 2H), 3.66–3.58 (m, 2H), 3.56–3.45 (m, 2H), 2.93–2.77 (m, 4H), 2.69–2.52 (m, 2H), 2.10–2.01 (m, 2H), 1.96–1.84 (m, 2H), 1.50 (s, 3H), 1.44 (s, 3H), 1.06 (d, *J* = 6.4 Hz,3H), 1.03 (d, *J* = 6.4 Hz, 3H). ^13^C NMR (101 MHz, CDCl_3_) δ 170.61, 161.50, 154.68, 154.62, 151.34, 130.72, 129.83, 115.48, 109.76, 109.55, 108.44, 108.32, 99.55, 97.90, 96.72, 74.31, 74.23, 47.62, 47.37, 40.87, 35.46, 35.29, 34.55, 23.02, 22.83, 18.53, 18.35, 15.98, 15.86. HR-EI-MS *m/z* 509.2404; calculated for C_29_H_35_NO_7_: 509.2414.

**Compound 6**. The title compound was obtained from xyloketal B acid and glycine methyl ester hydrochloride with a procedure similar to that for compound **1**. Yield 78%; white solid; m. p. 75–76 °C. ^1^H NMR (400 MHz, CDCl_3_) δ 13.97 (s, 1H), 9.03 (s, 1H), 4.28–4.21 (m, 2H), 4.19–4.11 (m, 2H), 3.78 (s, 3H), 3.56–3.48 (m, 2H), 2.91–2.81 (m, 2H), 2.67–2.56 (m, 2H), 2.15–2.04 (m, 2H), 1.96–1.88 (m, 2H), 1.60 (s, 3H), 1.50 (s, 3H), 1.06 (d, *J* = 6.4 Hz, 3H), 1.04 (d, *J* = 6.4 Hz, 3H). ^13^C NMR (101 MHz, CDCl_3_) δ 170.57, 170.12, 161.48, 154.93, 151.52, 109.75, 108.40, 99.66, 98.28, 96.51, 74.41, 74.17, 52.28, 47.76, 47.27, 41.31, 35.70, 35.22, 22.96, 22.75, 18.60, 18.30, 16.04, 15.85. HR-EI-MS *m/z* 461.2043; calculated for C_24_H_31_NO_8_: 461.2050.

**Compound 7**. The title compound was obtained from xyloketal B acid and valine methyl ester hydrochloride with a procedure similar to that for compound **1**. Yield 76%; white solid; m. p. 80–81 °C. ^1^H NMR (400 MHz, CDCl_3_) δ 14.08 (s, 1H), 9.17 (s, 1H), 4.68–4.62 (m, 1H), 4.25–4.08 (m, 2H), 3.77 (s, 3H), 3.59–3.51 (m, 2H), 2.94–2.86 (m, 2H), 2.70–2.57 (m, 2H), 2.34–2.28 (m, 1H), 2.22–2.07 (m, 2H), 2.01–1.93 (m, 2H), 1.62 (s, 3H), 1.54 (s, 3H), 1.09 (d, *J* = 6.4 Hz, 3H), 1.07 (d, *J* = 6.4 Hz, 3H), 1.04 (d, *J* = 6.8 Hz, 6H). ^13^C NMR (101 MHz, CDCl_3_) δ 172.19, 170.47, 161.57, 154.90, 151.53, 109.73, 108.42, 99.49, 98.22, 96.62, 77.02, 74.37, 74.26, 57.23, 52.04, 47.53, 47.33, 35.27, 30.92, 23.20, 23.01, 22.76, 19.22, 18.66, 18.36, 17.54, 16.23, 15.88. HR-EI-MS *m/z* 503.2515; calculated for C_27_H_37_NO_8_: 503.2519.

**Compound 8**. The title compound was obtained from xyloketal B acid and tyrosine methyl ester hydrochloride with a procedure similar to that for compound **1**. Yield 75%; white solid; m. p. 95–95 °C. ^1^H NMR (400 MHz, CDCl_3_) δ 14.06 (s, 1H), 9.01 (s, 1H), 7.04 (d, *J* = 8.0 Hz, 2H), 6.71 (d, *J* = 8.0 Hz, 2H), 5.5 (br, 1H), 4.92–4.87 (m, 1H), 4.22–3.97 (m, 2H), 3.72 (s, 3H), 3.57–3.48 (m, 2H), 3.21–3.15 (m, 1H), 3.04 (d, *J* = 6.8 Hz, 1H), 2.90–2.77 (m, 2H), 2.66–2.53 (m, 2H), 2.11–2.02 (m, 2H), 1.96–1.85 (m, 2H), 1.51 (s, 3H), 1.39 (s, 3H), 1.06 (d, *J* = 6.4 Hz, 3H), 0.98 (d, *J* = 6.4 Hz, 3H). ^13^C NMR (101 MHz, CDCl_3_) δ 171.99, 170.10, 161.58, 154.98, 154.82, 151.63, 130.48, 128.23, 115.46, 109.82, 109.59, 108.44, 108.33, 99.34, 97.83, 96.40, 74.25, 74.09, 54.13, 52.29, 47.56, 47.29, 37.24, 35.23, 23.04, 22.79, 19.16, 18.72, 18.30, 16.16, 15.99. HR-EI-MS *m/z* 567.2465; calculated for C_31_H_37_NO_9_: 567.2468.

**Compound 9**. The title compound was obtained from xyloketal B acid and cysteamine with a procedure similar to that for compound **1**. Yield 73%; white solid; m. p. 72–73 °C. ^1^H NMR (400 MHz, CDCl_3_) δ 14.26 (s, 1H), 8.84 (s, 1H), 4.21–4.13 (m, 2H), 3.80–3.70 (m, 2H), 3.57–3.48 (m, 2H), 2.90 (t, *J* = 6.4 Hz, 2H), 2.86–2.80 (m, 2H), 2.70–2.56 (m, 2H), 2.11–2.03 (m, 2H), 1.97–1.90 (m, 2H), 1.67 (br, 1H), 1.55 (s, 3H), 1.51 (s, 3H), 1.07 (d, *J* = 6.4 Hz, 3H), 1.04 (d, *J* = 6.4 Hz, 3H). ^13^C NMR (101 MHz, CDCl_3_) δ 170.72, 161.59, 154.98, 151.45, 109.66, 108.27, 99.47, 97.80, 96.64, 74.42, 74.22, 47.65, 47.33, 38.06, 37.76, 35.62, 35.25, 23.22, 22.93, 18.60, 18.31, 16.04, 15.90. HR-EI-MS *m/z* 449.1869; calculated for C_23_H_31_NO_6_S: 449.1872.

**Compound 10**. The title compound was obtained from xyloketal B acid and *N*,*N*-dimethyl ethylenediamine with a procedure similar to that for compound **1**. Yield 70%; white solid; m. p. 71–72 °C. ^1^H NMR (400 MHz, CDCl_3_) δ 13.49 (s, 1H), 8.94 (s, 1H), 4.17 (dd, *J* = 16.4, 6.8 Hz, 2H), 3.75–3.65 (m, 2H), 3.52 (q, *J* = 6.8 Hz, 2H), 3.27 (t, *J* = 5.6 Hz, 2H), 2.91 (s, 6H), 2.82 (d, *J* = 6.8 Hz, 2H), 2.60–2.49 (m, 2H), 2.07–2.01 (m, 2H), 1.96–1.88 (m, 2H), 1.53 (s, 3H), 1.50 (s, 3H), 1.06 (d, *J* = 6.8 Hz, 3H), 1.02 (d, *J* = 6.8 Hz, 3H). ^13^C NMR (101 MHz, CDCl_3_) δ 172.26, 161.34, 155.71, 151.49, 110.03, 108.58, 99.59, 98.46, 95.89, 74.37, 74.18, 59.20, 47.46, 47.18, 44.18, 36.69, 36.01, 35.40, 23.04, 22.75, 18.36, 18.25, 15.95, 15.81, 15.68. HR-EI-MS *m/z* 460.2563; calculated for C_25_H_36_N_2_O_6_: 460.2573.

**Compound 11**. The title compound was obtained from xyloketal B acid and thiophene ethylamine with a procedure similar to that for compound **1**. Yield 76%; white solid; m. p. 75–76 °C. ^1^H NMR (400 MHz, CDCl_3_) δ 14.41 (s, 1H), 8.66 (s, 1H), 7.14 (d, *J* = 5.2 Hz, 1H), 6.92 (d, *J* = 5.2 Hz, 1H), 6.90–6.89 (m, 1H), 4.25 (dd, *J* = 16.4, 6.8 Hz, 2H), 3.76–3.63 (m, 2H), 3.56–3.45 (m, 2H), 3.13 (t, *J* = 6.8 Hz, 2H), 2.93–2.79 (m, 2H), 2.69–2.53 (m, 2H), 2.11–2.00 (m, 2H), 1.97–1.86 (m, 2H), 1.51 (s, 3H), 1.42 (s, 3H), 1.07 (d, *J* = 6.4 Hz, 3H), 1.03 (d, *J* = 6.4 Hz, 3H). ^13^C NMR (101 MHz, CDCl_3_) δ 170.67, 161.65, 154.67, 151.35, 141.59, 126.94, 125.34, 123.73, 109.46, 108.37, 99.45, 97.56, 96.64, 74.34, 74.21, 47.56, 47.34, 40.70, 35.53, 35.26, 29.80, 22.97, 22.68, 18.67, 18.38, 16.06, 15.91. HR-EI-MS *m/z* 499.2025; calculated for C_27_H_33_NO_6_S: 499.2029.

**Compound 12**. The title compound was obtained from xyloketal B acid and phenylalanine methyl ester hydrochloride with a procedure similar to that for compound **1**. Yield 73%; white solid; m. p. 72–73 °C. ^1^H NMR (400 MHz, CDCl_3_) δ 14.05 (s, 1H), 9.03(s, 1H), 7.27–7.20 (m, 5H), 5.00–4.95 (m, 1H), 4.21–4.15 (m, 2H), 3.74 (s, 3H), 3.59–3.50 (m, 2H), 3.32–3.26 (m, 1H), 3.14 (d, *J* = 7.2 Hz, 1H), 2.93–2.80 (m, 2H), 2.73–2.54 (m, 2H), 2.11–2.05 (m, 2H), 1.97–1.90 (m, 2H), 1.52 (s, 3H), 1.38 (s, 3H), 1.08 (d, *J* = 6.4 Hz, 3H), 1.05 (d, *J* = 6.4 Hz, 3H). ^13^C NMR (101 MHz, CDCl3) δ 171.71, 170.14, 161.63, 154.96, 151.66, 136.47, 129.31, 128.49, 128.39, 126.79, 109.78, 109.57, 108.41, 99.34, 97.89, 96.42, 74.30, 74.18, 53.77, 52.16, 47.62, 47.37, 38.04, 35.66, 22.93, 22.66, 18.71, 18.64, 18.28, 16.04, 15.87. HR-EI-MS *m/z* 551.2512; calculated for C_31_H_37_NO_8_: 551.2519.

**Compound 13**. The title compound was obtained from xyloketal B acid and tryptophan methyl hydrochloride with a procedure similar to that for compound **1**. Yield 72%; white solid; m. p. 110–111 °C. ^1^H NMR (400 MHz, CDCl_3_) δ 14.14 (s, 1H), 9.03 (s, 1H), 8.19 (s, 1H), 7.55 (t, *J* = 8.0 Hz, 1H), 7.30 (d, *J* = 8.0 Hz, 1H), 7.17(s, 1H), 7.13–7.11 (m, 1H), 7.06–7.00 (m, 1H), 5.10–4.99 (m, 1H), 4.15 (dd, *J* = 16.4, 6.8 Hz, 2H), 3.67 (s, 3H), 3.57–3.45 (m, 2H), 3.40–3.29 (m, 2H), 2.92–2.77 (m, 2H), 2.70–2.55 (m, 2H), 2.11–2.01 (m, 2H), 1.89–1.82 (m, 2H), 1.52 (s, 3H), 1.48 (s, 3H), 1.06 (d, *J* = 6.4 Hz, 3H), 1.02 (d, *J* = 6.4 Hz, 3H). ^13^C NMR (101 MHz, CDCl_3_) δ 172.18, 170.12, 161.56, 154.99, 151.52, 136.11, 127.63, 123.18, 121.81, 119.42, 118.75, 110.95, 110.20, 109.54, 108.34, 99.26, 97.71, 96.46, 74.18, 74.00, 53.34, 52.19, 47.43, 47.24, 35.44, 35.15, 27.72, 22.91, 22.71, 22.40, 18.64, 18.26, 15.82. HR-EI-MS *m/z* 590.2623; calculated for C_33_H_38_N_2_O_8_: 590.2628.

**Compound 16**. First, 50 mg (0.14 mmol) of xyloketal B [16] and 8.7 mg (0.28 mmol) of methylamine were dissolved in 10 mL of DMF and stirred, followed by adding 0.05 mL (0.28 mmol) of 40% formaldehyde solution, stirred at room temperature for 1 h. The reaction was quenched with water. The aqueous layer was extracted with ethyl acetate, 3 × 25 mL. The combined organic extracts were washed with saturated ammonium chloride and brine, dried over anhydrous MgSO_4_ and concentrated *in vacuo*. Purification by flash chromatography (petroleum ether: ethyl acetate = 5:1~2:1) gave the title compound. Yield 89%; white solid; m. p. 62–63 °C. ^1^H NMR (400 MHz, CDCl_3_) δ 4.66 (s, 2H), 4.15–4.08 (m, 2H), 3.75 (s, 2H), 3.49 (t, *J* = 8.4 Hz, 2H), 2.79 (dt, *J* = 17.2 5.2 Hz, 2H), 2.60 (d, *J* = 17.2 Hz, 2H), 2.53 (s, 3H), 2.11–2.04 (m, 2H), 1.87–1.81 (m, 2H), 1.47 (s, 3H), 1.44 (s, 3H), 1.04 (d, *J* = 6.4 Hz, 3H), 1.01 (d, *J* = 6.4 Hz, 3H); ^13^C NMR (101 MHz, CDCl_3_): δ 150.13, 149.50, 148.87, 107.25, 107.14, 100.01, 98.73, 98.12, 83.58, 73.85, 47.33, 47.47, 40.03, 35.42, 35.32, 22.98, 22.72, 18.73, 18.39, 16.07, 15.99. HR-EI-MS *m/z* 401.2196; calculated for C_23_H_31_O_5_N_1_: 401.2197.

**Compound 17**. The title compound was obtained from xyloketal B and butylamine with a procedure similar to that for compound **16**. Yield 93%; white solid; m. p. 65–66 °C. ^1^H NMR (400 MHz, CDCl_3_) δ 4.76 (s, 2H), 4.20–4.12 (m, 2H), 3.82 (s, 2H), 3.52 (t, *J* = 8.4 Hz, 2H), 2.86–2.80 (m, 2H), 2.69 (t, *J* = 17.2 Hz, 2H), 2.67–2.59 (m, 2H), 2.17–2.11 (m, 2H), 1.91–1.83 (m, 2H), 1.58–1.51 (m, 2H), 1.49 (s, 3H), 1.48 (s, 3H), 1.00–1.30 (m, 2H), 1.06 (d, *J* = 6.4 Hz, 3H), 1.03 (d, *J* = 6.4 Hz, 3H), 0.92 (t, *J* = 7.6 Hz, 3H); ^13^C NMR (101 MHz, CDCl_3_) δ 150.75, 149.40, 148.95, 107.33, 107.24, 100.49, 98.72, 98.25, 82.68, 73.96, 51.64, 47.59, 47.59, 45.91, 35.55, 35.44, 30.29, 23.06, 22.79, 20.48, 18.85, 18.506, 16.081, 16.00, 14.05. HR-EI-MS *m/z* 443.2665; calculated for C_26_H_37_O_5_N_1_: 443.2666.

**Compound 18**. The title compound was obtained from xyloketal B and ethanolamine with a procedure similar to that for compound **16**. Yield 79%; white solid; m. p. 112–113 °C. ^1^H NMR (400 MHz, CDCl_3_) δ 4.80 (s, 2H), 4.2 (s, 1H), 4.16 (dd, *J* = 16.4, 6.4 Hz, 2H), 3.86 (s, 2H), 3.68 (t, *J* = 5.2 Hz, 2H), 3.54–3.47 (m, 2H), 2.92–2.89 (m, 2H), 2.86–2.75 (m, 2H), 2.66–2.59 (m, 2H), 2.15–2.07 (m, 2H), 1.91–1.82 (m, 2H), 1.49 (s, 3H), 1.47 (s, 3H), 1.06 (d, *J* = 6.4 Hz, 3H), 1.03 (d, *J* = 6.4 Hz, 3H); ^13^C NMR (CDCl_3_, 101 MHz) δ 150.55, 149.64, 148.83, 107.27, 99.91, 98.82, 98.21, 82.79, 73.90, 59.02, 53.77, 47.36, 47.26, 45.39, 35.31, 22.93, 22.67, 18.63, 18.38, 15.95. HR-EI-MS *m/z* 431.2301; calculated for C_24_H_33_O_6_N_1_: 431.2302.

**Compound 19**. The title compound was obtained from xyloketal B and diglycolamine with a procedure similar to that for compound **16**. Yield 72%; white solid; m. p. 126–127 °C. ^1^H NMR (400 MHz, CDCl_3_) δ 4.82 (s, 2H), 4.14 (dd, *J* = 16.4, 6.4 Hz, 2H), 3.89 (s, 2H), 3.73 (t, *J* = 5.2 Hz, 2H), 3.68 (t, *J* = 5.2 Hz, 2H), 3.61 (t, *J* = 5.2 Hz, 2H), 3.55–3.48 (m, 2H), 2.97 (br, 1H), 2.95–2.90 (m, 2H), 2.86–2.75 (m, 2H), 2.62 (t, *J* = 5.6 Hz, 2H), 2.16–2.06 (m, 2H), 1.91–1.84 (m, 2H), 1.50 (s, 3H), 1.47 (s, 3H), 1.06 (d, *J* = 6.4 Hz, 3H), 1.03 (d, *J* = 6.4 Hz, 3H); ^13^C NMR (101 MHz, CDCl_3_) δ 150.55, 149.68, 148.98, 107.39, 107.22, 99.93, 99.01, 98.31, 82.66, 73.96, 72.39, 69.21, 61.70, 51.59, 47.58, 47.42, 46.05, 35.51, 35.37, 23.02, 22.75, 18.76, 18.37, 16.01, 15.91. HR-EI-MS *m/z* 475.2563; calculated for C_26_H_37_O_7_N_1_: 475.2565.

**Compound 20**. The title compound was obtained from xyloketal B and aniline with a procedure similar to that for compound **16**. Yield 93%; white solid; m. p. 68–69 °C. ^1^H NMR (400 MHz, CDCl_3_) δ 7.25 (t, *J* = 8.8 Hz, 2H), 7.10 (d, *J* = 8.8 Hz, 2H), 6.88 (t, *J* = 8.0 Hz, 1H), 5.35–5.25 (m, 2H), 4.46 (s, 2H), 4.14 (dd, *J* = 17.2, 8.0 Hz, 2H), 3.49 (dd, *J* = 17.2, 8.0 Hz, 2H), 2.79 (t, *J* = 17.2 Hz, 2H), 2.65–2.58 (m, 2H), 2.11–2.05 (m, 2H), 1.88–1.83 (m, 2H), 1.48 (s, 3H), 1.46 (s, 3H), 1.04 (d, *J* = 6.4 Hz, 3H), 1.01 (d, *J* = 6.4 Hz, 3H); ^13^C NMR (CDCl_3_, 101 MHz) δ 151.05, 149.86, 148.38, 148.38, 129.03, 120.59, 117.51, 107.46, 107.29, 101.23, 99.05, 96.66, 78.84, 73.99, 47.59, 47.46, 46.34, 35.41, 23.04, 22.75, 18.81, 18.73, 18.50, 16.06, 15.97. HR-EI-MS *m/z* 463.2352; calculated for C_28_H_33_O_5_N_1_: 463.2353.

**Compound 21**. The title compound was obtained from xyloketal B and *p*-methoxyaniline with a procedure similar to that for compound **16**. Yield 89%; white solid; m. p. 89–90 °C. ^1^H NMR (400 MHz, CDCl_3_) δ 7.01 (d, *J* = 9.2 Hz, 2H), 6.78 (d, *J* = 9.2 Hz, 2H), 5.25–5.17 (m, 2H), 4.38 (s, 2H), 4.13 (dd, *J* = 17.2, 8.0 Hz, 2H), 3.73 (s, 3H), 3.49 (dd, *J* = 17.2, 8.0 Hz, 2H), 2.78 (t, *J* = 17.2 Hz, 2H), 2.64–2.56 (m, 2H), 2.10–2.06 (m, 2H), 1.87–1.81 (m, 2H), 1.51 (s, 3H), 1.46 (s, 3H), 1.03 (d, *J* = 6.8 Hz, 3H), 1.00 (d, *J* = 6.8 Hz, 3H); ^13^C NMR (CDCl_3_, 101 MHz) δ 154.33, 150.96, 149.83, 148.38, 142.55, 119.87, 114.35, 107.43, 107.28, 101.14, 98.99, 98.62, 80.10, 73.99, 55.53, 47.57, 47.49, 47.036, 35.432, 23.06, 22.79, 18.74, 18.47, 16.08, 15.98. HR-EI-MS *m/z* 493.2458; calculated for C_29_H_35_O_6_N_1_: 493.2459.

**Compound 22**. The title compound was obtained from xyloketal B and 4-aminophenol with a procedure similar to that for compound **16**. Yield 85%; white solid; m. p. 102–103 °C. ^1^H NMR (400 MHz, CDCl_3_) δ 6.99 (d, *J* = 8.8 Hz, 2H), 6.74 (d, *J* = 8.8 Hz, 2H), 5.25–5.16 (m, 2H), 4.37 (s, 2H), 4.14 (dd, *J* = 17.2, 8.0 Hz, 2H), 3.51 (dd, *J* = 17.2, 8.0 Hz, 2H), 2.79 (t, *J* = 17.2 Hz, 2H), 2.66–2.58 (m, 2H), 2.12–2.04 (m, 2H), 1.90–1.84 (m, 2H), 1.47 (s, 6H), 1.04 (d, *J* = 6.4 Hz, 3H), 1.01 (d, *J* = 6.4 Hz, 3H); ^13^C NMR (CDCl_3_, 101 MHz) δ 150.91, 150.78, 149.73, 148.24, 142.18, 120.16, 115.77, 109.64, 107.53, 107.36, 101.20, 99.00, 98.76, 80.23, 73.98, 47.53, 47.47, 47.07, 35.39, 23.08, 22.84, 18.69, 18.42, 16.29, 15.98. HR-EI-MS *m/z* 479.2308; calculated for C_28_H_33_O_6_N_1_: 479.2309.

**Compound 23**. The title compound was obtained from xyloketal B and *p*-aminobenzoic with a procedure similar to that for compound **16**. Yield 88%; white solid; m. p. 128–129 °C. ^1^H NMR (400 MHz, CDCl_3_) δ 12.58 (s, 1H), 8.01 (d, *J* = 8.8 Hz, 2H), 7.08 (d, *J* = 8.8 Hz, 2H), 5.42–5.32 (m, 2H), 4.54 (s, 2H), 4.16 (dd, *J* = 17.2, 8.0 Hz, 2H), 3.55–3.49 (m, 2H), 2.81 (t, *J* = 17.2 Hz, 2H), 2.68–2.60 (m, 2H), 2. 11–2.06 (m, 2H), 1.90–1.86 (m, 2H), 1.51 (s, 3H), 1.48 (s, 3H), 1.05 (d, *J* = 6.8 Hz, 3H), 1.03 (d, *J* = 6.8 Hz, 3H); ^13^C NMR (CDCl_3_, 101 MHz) δ 171.51, 152.21, 150.86, 149.97, 148.25, 131.82, 120.05, 114.94, 107.54, 107.40, 100.82, 99.38, 98.68, 73.97, 47.39, 47.21, 45.53, 35.29, 22.96, 22.71, 18.71, 18.39, 16.01, 15.95. HR-EI-MS *m/z* 507.2253; calculated for C_29_H_33_O_7_N_1_: 507.2252.

**Compound 24**. The title compound was obtained from xyloketal B and paranitroaniline with a procedure similar to that for compound **16**. Yield 82%; white solid; m. p. 70–71 °C. ^1^H NMR (400 MHz, CDCl_3_) δ 8.14 (d, *J* = 9.2 Hz, 2H), 7.06 (d, *J* = 9.2 Hz, 2H), 5.42–5.33 (m, 2H), 4.56 (s, 2H), 4.17 (dd, *J* = 17.2, 8.0 Hz, 2H), 3.55–3.50 (m, 2H), 2.82 (t, *J* = 17.2 Hz, 2H), 2.68–2.60 (m, 2H), 2.13–2.04 (m, 2H), 1.92–1.88 (m, 2H), 1.51 (s, 3H), 1.49 (s, 3H), 1.06 (d, *J* = 6.8 Hz, 3H), 1.04 (d, *J* = 6.8 Hz, 3H); ^13^C NMR (CDCl_3,_ 101 MHz) δ 153.00, 150.90, 150.47, 150.31, 148.56, 139.93, 125.74, 114.46, 107.81, 107.64, 100.47, 99.98, 99.85, 98.99, 76.57, 73.97, 47.57, 47.37, 45.49, 35.46, 35.34, 23.02, 22.78, 18.63, 18.34, 15.99, 15.86. HR-EI-MS *m/z* 508.2205; calculated for C_28_H_32_O_7_N_2_: 508.2204.

**Compound 25**. The title compound was obtained from xyloketal B and *p*-fluoro aniline with a procedure similar to that for compound **16**. Yield 87%; white solid; m. p. 79–80 °C. ^1^H NMR (400 MHz, CDCl_3_) δ 7.07 (d, *J* = 9.2 Hz, 2H), 6.95 (d, *J* = 9.2 Hz, 1H), 6.93 (d, *J* = 9.2 Hz, 1H), 5.24 (s, 2H), 4.42 (s, 2H), 4.15 (dd, *J* = 17.2, 8.0 Hz, 2H), 3.49 (dd, *J* = 17.2, 8.0 Hz, 2H), 2.79 (t, *J* = 17.2 Hz, 2H), 2.67–2.58 (m, 2H), 2.14–2.03 (m, 2H), 1.90–1.84 (m, 2H), 1.48 (s, 6H), 1.05 (d, *J* = 6.8 Hz, 3H), 1.02 (d, *J* = 6.8 Hz, 3H); ^13^C NMR (101 MHz, CDCl_3_) δ 159.24, 156.05, 150.85, 149.89, 148.28, 144.96, 144.817, 119.78, 119.55, 115.62, 115.34, 107.46, 107.28, 100.84, 99.14, 98.64, 79.52, 73.94, 70.38, 47.55, 46.99, 35.50, 35.39, 23.04, 22.77, 18.76, 18.41, 16.02. HR-EI-MS *m/z* 481.2259; calculated for C_28_H_32_O_5_N_1_F_1_: 481.2259.

**Compound 26**. The title compound was obtained from xyloketal B and parachloroaniline with a procedure similar to that for compound **16**. Yield 78%; white solid; m. p. 82–83 °C. ^1^H NMR (400 MHz, CDCl_3_) δ 7.20 (d, *J* = 8.4 Hz, 2H), 7.03 (d, *J* = 8.4 Hz, 2H), 5.31–5.22 (m, 2H), 4.43 (s, 2H), 4.15 (dd, *J* = 17.2, 8.4 Hz, 2H), 3.52 (dd, *J* = 17.2, 8.0 Hz, 2H), 2.79 (t, *J* = 17.2 Hz, 2H), 2.66–2.58 (m, 2H), 2.13–2.03 (m, 2H), 1.90–1.85 (m, 2H), 1.48 (s, 6H), 1.47 (s, 3H), 1.05 (d, *J* = 6.8 Hz, 3H), 1.02 (d, *J* = 6.8 Hz, 3H); ^13^C NMR (101MHz, CDCl_3_) δ 150.84, 149.96, 149.81, 148.34, 147.08, 128.90, 125.55, 118.85, 107.52, 107.32, 100..82, 99.24, 98.67, 78.73, 73.98, 47.56, 47.40, 46.54, 35.40, 23.05, 22.78, 18.70, 18.43, 16.04. HR-EI-MS *m/z* 497.1965; calculated for C_28_H_32_O_5_N_1_Cl_1_: 497.1964.

**Compound 27**. The title compound was obtained from xyloketal B and parabromoaniline with a procedure similar to that for compound **16**. Yield 88%; white solid; m. p. 83–84 °C. ^1^H NMR (400 MHz, CDCl_3_) δ 7.34 (d, *J* = 9.2 Hz, 2H), 6.98 (d, *J* = 9.2 Hz, 2H), 5.31–5.22 (m, 2H), 4.43 (s, 2H), 4.15 (dd, *J* = 17.2, 8.0 Hz, 2H), 3.51 (dd, *J* = 17.2, 8.0 Hz, 2H), 2.79 (t, *J* = 17.2 Hz, 2H), 2.64–2.56 (m, 2H), 2.10–2.01 (m, 2H), 1.88–1.83 (m, 2H), 1.48 (s, 6H), 1.05 (d, *J* = 6.8 Hz, 3H), 1.02 (d, *J* = 6.8 Hz, 3H); ^13^C NMR (101 MHz, CDCl_3_) δ 150.82, 149.96, 149.80, 148.33, 147.51, 131.81, 119.21, 112.89, 107.51, 107.31, 100.79, 99.24, 98.66, 78.56, 73.97, 47.55, 47.40, 46.44, 35.50, 35.39, 23.04, 22.79, 18.70, 18.46, 16.04, 15.93. HR-EI-MS *m/z* 541.1455; calculated for C_28_H_32_O_5_N_1_Br_1_: 541.1458.

**Compound 28**. The title compound was obtained from xyloketal B and paraiodoaniline with a procedure similar to that for compound **16**. Yield 86%; white solid; m. p. 85–86 °C. ^1^H NMR (400 MHz, CDCl_3_) δ 7.50 (d, *J* = 8.8 Hz, 2H), 6.85 (d, *J* = 8.8 Hz, 2H), 5.29–5.20 (m, 2H), 4.41 (s, 2H), 4.13 (dd, *J* = 17.2, 8.4 Hz, 2H), 3.50 (dd, *J* = 17.2, 8.4 Hz, 2H), 2.77 (t, *J* = 17.2 Hz, 2H), 2.63–2.59 (m, 2H), 2.9–2.01 (m, 2H), 1.87–1.82 (m, 2H), 1.46 (s, 6H), 1.03 (d, *J* = 6.8 Hz, 3H), 1.01 (d, *J* = 6.8 Hz, 3H); ^13^C NMR (101 MHz, CDCl_3_) δ 150.82, 149.97, 149.82, 148.34, 148.14, 137.76, 119.62, 107.57, 107.32, 100.82, 99.25, 98.67, 82.75, 78.36, 73.99, 47.55, 47.39, 46.32, 35.39, 23.06, 22.77, 18.69, 18.42, 16.05, 15.94. HR-EI-MS *m/z* 589.1312; calculated for C_28_H_32_O_5_N_1_I_1_: 589.1320.

**Compound 29**. First, 50 mg (0.14 mmol) of xyloketal B [16] and 24 mg (0.28 mmol) of morpholine were dissolved in 10 mL of DMF and stirred, followed by adding 0.05 mL (0.28 mmol) of 40% formaldehyde solution, stirred at room temperature for 1 h. The reaction was quenched with water. The aqueous layer was extracted with ethyl acetate, 3 × 25 mL. The combined organic extracts were washed with saturated ammonium chloride and brine, dried over anhydrous MgSO_4_ and concentrated *in vacuo*. Purification by flash chromatography (petroleum ether: ethyl acetate = 5:1~2:1) gave the title compound. Yield 81%; white solid; m. p. 80–81 °C. ^1^H NMR (400 MHz, CDCl_3_) δ 4.12–4.03 (m, 2H), 3.77–3.67 (m, 4H), 3.71 (s, 2H), 3.44 (q, *J* = 8.8 Hz, 2H), 2.77 (t, *J* = 17.2 Hz, 2H), 2.66–2.62 (m, 2H), 2.61–2.52 (m, 4H), 2.11–1.98 (m, 2H), 1.89–1.79 (m, 2H), 1.47 (s, 3H), 1.43 (s, 3H), 1.03 (d, *J* = 8.4 Hz, 3H), 0.99 (d, *J* = 8.4 Hz, 3H); ^13^C NMR (CDCl_3_, 101 MHz) δ 155.18, 150.84, 150.03, 108.00, 107.77, 100.04, 98.83, 98.59, 74.26, 67.16, 54.62, 53.11, 48.19, 47.97, 36.16, 35.82, 23.52, 23.16, 19.35, 19.03, 16.54, 16.44. HR-EI-MS *m/z* 445.2458; calculated for C_25_H_35_O_6_N_1_: 445.2459.

**Compound 30**. The title compound was obtained from xyloketal B and diethylamine with a procedure similar to that for compound **29**. Yield 81%; white solid; m. p. 94–95 °C. ^1^H NMR (400 MHz, CDCl_3_) δ 4.15 (dd, *J* = 17.2, 8.4 Hz, 2H), 3.78 (q, *J* = 11.2 Hz, 2H), 3.49 (dd, *J* = 17.2, 8.4 Hz, 2H), 2.86–2.80 (m, 2H), 2.70–2.64 (m, 2H), 2.64–2.58 (m, 4H), 2.12–2.03 (m, 2H), 1.92–1.83 (m, 2H), 1.50 (s, 6H), 1.09 (t, 6H), 1.06 (d, *J* = 6.8 Hz, 3H), 1.03 (d, *J* = 6.8 Hz, 3H); ^13^C NMR (CDCl_3_, 101 MHz) δ 155.76, 150.29, 149.37, 107.45, 107.32, 100.76, 98.42, 97.50, 73.93, 49.36, 47.62, 46.31, 35.71, 35.46, 23.13, 22.83, 18.70, 16.07, 11.21. HR-EI-MS *m/z* 431.2668; calculated for C_25_H_37_O_5_N_1_: 431.2666.

### 3.3. Biological Evaluation

#### 3.3.1. Pharmacological Assays

Xyloketals derivatives were obtained using the above synthetic method; DMEM (High Glucose) and FBS were purchased from Gibco BRL (Grand Island, NY, USA); JC-1 probe was purchased from Beyotime Institute of Biotechnology (Haimen, China); H_2_O_2_ was purchased from Guangzhou Chemical Reagent Factory (GCRF, Guangzhou, China) and was freshly prepared for each experiment from a 30% stock solution. All other reagents were purchased from Sigma (St. Louis, MO, USA).

The HUVECs cell line was provided by the Pharmaceutical Biotechnology Centre of Jinan University (Guangdong, China). The cells were cultured in a DMEM medium (High Glucose) (Gibco, Grand Island, NY, USA) supplemented with 10% fetal bovine serum (FBS) (Gibco, Grand Island, NY, USA), penicillin (100 U/mL) and streptomycin (100 U/mL) at 37 °C in a 5% CO_2_ humidified incubator. Endothelial cells appear as “cobblestone” mosaic after reaching confluence under a microscope.

HUVECs were harvested during the logarithmic growth phase and seeded in 96-well plates at a density of 6 × 10^4^/mL and cultured at 37 °C in a 5% CO_2_ humidified incubator for 24 h. The cell viability was assessed using the mitochondrial tetrazolium assay (MTT) in HUVECs. The cells were pre-incubated with xyloketals at different concentrations (10 μM, 1 μM) for 30 min, followed by exposure to H_2_O_2_ at a concentration of 600 μM and additional incubation for 20 h. MTT solution (15 μL/well, 5 μg/mL) was added and processed to examine the cell viability. The optical density was read at λ = 570 nm using a Thermo Multiskan FC plate reader. At the tested concentration, all of the xyloketals showed no significant effects on cell viability.

#### 3.3.2. Construction and Validation of the QSAR Model

The three-dimensional structures of the compounds were constructed using the SYBYL programming package (version 7.3.5, Tripos, St. Louis, MO, USA). The MMFF94 force field and MMFF94 partial atomic charges were applied to these compounds. In addition, the compounds were minimized using a non-bond cut-off of 8 angstroms and the Powell conjugate-gradient algorithm. The convergence criterion was set to 0.05 kcal/mol. The activities of the compounds at 10 μM were expressed using the LOGIT transformation shown in the following formula to give a value in proportion to energy: $\text{LOGIT}=-\text{ log }(\text{tested concentration})+\text{log}\frac{\text{\%cell viability}}{100\;-\text{\%cell viability}}$

The training set and test set were randomly divided out of a total of 35 molecules. A training set of 30 molecules was used to construct the QSAR model. In addition, a training set of 5 molecules was used to validate it. All of the molecules were aligned using the most active compound, **22**, as the template. Each compound was mapped onto a 3D lattice with grid points 2.0 Å apart. The mapped region was created automatically by the program with an attenuation factor of 0.3. The electrostatic, hydrophobic, donor and acceptor columns were used to construct the model. The model was constructed using the partial-least-squares (PLS) analysis without any column filtering.

The robustness of the model was addressed based on the internal cross-validation using the leave-one-out (LOO) procedure and the external validation of the test set. All of the statistical parameters are listed in Table 3.

#### 3.3.3. Mitochondrial Membrane Potentials Assay

The JC-1 probe was used to measure the mitochondrial depolarization in HUVECs. Briefly, HUVECs were cultured in six-well plates at a density of 2.5 × 10^5^/mL, incubated with **23** and **24** at different concentrations (25 μM and10 μM) for 30 min, then exposed to 400 μM H_2_O_2_ for 20 h. According to the instructions for the test kits, all cells were collected into 1.5-mL tubes, incubated with JC-1 for 20 min at 37 °C and rinsed twice with PBS. The mitochondrial membrane potentials (ΔΨm) were monitored by determining the relative amounts of dual emissions from JC-1 monomers or aggregates using a BD FACS Aria flow cytometry. The red/green fluorescence was calculated using the Flowjo (v7.6.5, Ashland, OR, USA).

#### 3.3.4. Statistics

Results are presented as mean ± S.E.M. Comparisons between multiple groups were performed using the Excel by *t*-test. Differences were considered to be significant at *p* ≤ 0.05.

## 4. Conclusions

The water insolubility of the xyloketal compounds from marine fungus may be challenging for further clinical development. Therefore, a new series of derivatives with the introduction of amino groups at the C-12 and C-13 positions of xyloketal B were designed and synthesized to improve the solubility and biological activity. All 28 new derivatives and seven known compounds (**14**, **15**, **31**–**35**) were evaluated for their protection against H_2_O_2_-induced HUVEC injury. The results indicated that some compounds exhibited strong anti-oxidative activities, especially compounds **23** and **24**, which displayed the best excellent protective activities out of all of the derivatives. Then, a CoMSIA was constructed using the SYBYL programming package (version 7.3.5) to explain the structural activity relationship of the xyloketal derivatives. A 3D QSAR model generated using the CoMSIA was analyzed and provided good advice to modify the molecules for better activity in the future. Compounds **23** and **24**, which had the most remarkable anti-oxidative activities, were further examined in the JC-1 mitochondrial membrane potential (MMP) assay of HUVECs. The results showed that compound **23** and **24** would significantly inhibit H_2_O_2_-induced the decrease in the cell mitochondrial membrane potential (ΔΨm) at 25 μM. In conclusion, we designed and synthesized a new series of xyloketal derivatives to improve solubility and biological activity. Among them, compounds **23** and **24** effectively protected HUVECs against oxidative damage and further mitochondrial membrane integrity impairment. These derivatives will be new candidates for the treatment of CVD.

## Abbreviations

3D-QSAR: three-dimensional quantitative structure-activity relationship
As: atherosclerosis
BOP: (benzotriazol-1-yloxy)tris(dimethylamino)phosphonium hexafluorophosphate
CoMSIA: a comparative molecular similarity indices analysis
CVD: cardiovascular disease
DCM: dichloromethane
DIEA: *N*,*N*-diisopropylethylamine
DMF: *N*,*N*-dimethylformamide
DMSO: dimethyl sulfoxide
FACs: fluorescence activating cell sorter
FCM: flow cytometry
H2DCFDA: 2′,7′-dihydrodichlorofluorescein diacetate
H2O2: hydrogen peroxide
HCHO: formaldehyde
HUVECs: human umbilical vein endothelial cells
LOO: leave-one-out
MAPK: mitogen-activated protein kinase
MMP: mitochondrial membrane potential
MPP+: 1-methyl-4-phenylpyridinium
NMR: nuclear magnetic resonance
OGD: oxygen-glucose deprivation
OxLDL: oxidized low density lipoprotein
PLS: partial-least-squares
ROS: reactive oxygen species
SAR: structure-activity relationship
THF: tetrahydrofuran
